# The effect of probiotics on the diarrhea and constipation outcomes in children: an umbrella review of systematic reviews and meta-analyses

**DOI:** 10.3389/fnut.2025.1606264

**Published:** 2025-07-18

**Authors:** Qizheng Wang, Tailiang Ren, Haijun Wang, Xiaofei Lin

**Affiliations:** ^1^Department of Pediatrics, Huai’an Maternal and Child Health Care Hospital Affiliated to Yangzhou University, Huai'an, China; ^2^Department of Pediatrics, The Huai'an Maternity and Child Clinical College of Xuzhou Medical University, Jiangsu, China; ^3^Department of Pediatrics, Lianshui County People’s Hospital, Affiliated Hospital of Kangda College, Nanjing Medical University, Jiangsu, China

**Keywords:** probiotics, diarrhea, constipation, systematic review, meta-analysis

## Abstract

**Background:**

The existing literature on the effects of probiotics on diarrhea and constipation outcomes remains inconsistent. Therefore, this umbrella review of systematic reviews and meta-analyses aims to provide a concise and definite understanding in relation to the effect of probiotics on diarrhea and constipation in children.

**Methods:**

A comprehensive systematic search was carried out in on Scopus, PubMed, Embase, Web of Science, and Google Scholar up to December 2024. The overall effect size was calculated using random effect model. Also, subgroup analyses were performed regarding age group, health condition, single or multi-strain probiotics.

**Results:**

This umbrella study comprises a systematic review of 35 studies. Our findings illustrated that probiotics reduce odds [odds ratio (OR) = 0.51; 95% confidence interval (CI): 0.27, 0.94] and risk of diarrhea incidence [relative risk (RR) = 0.54; 95% CI: 0.40, 0.71] compared to control group, meaningfully. Also, it is successful in reducing diarrhea duration [weighted mean difference (WMD) = −1.85; 95% CI: −2.83, −0.86] and [standardized mean difference (SMD) = −0.94; 95% CI: −1.32, −0.56] significantly. Moreover, probiotics supplementation resulted in decreased stool frequency (WMD = −0.21; 95% CI: −0.37, −0.04). Probiotics prevent diarrhea by about 36% (RR = 0.64; 95% CI: 0.63, 0.65(, and significantly improved diarrhea treatment (SMD = −0.49; 95% CI: −0.59, −0.38). Also, the analyses revealed that probiotics significantly impact on constipation (OR = 1.17, 95% CI: 1.01–1.37).

**Conclusion:**

This meta-analysis supports the potential role of probiotics in relation to diarrhea and constipation outcome in children. Probiotic supplementation contributed to a declined risk and odds of diarrhea incidence. Also, probiotic supplementation was accompanied with decreased diarrhea duration.

## Introduction

Gastrointestinal issues are fairly common throughout childhood and adulthood, affecting an estimated 8–25% of the general population (1). These percentages may vary based on the specific gastrointestinal condition, and the age of the individuals affected (2). Gastrointestinal disorders, including diarrhea and constipation, are common health concerns worldwide. Diarrhea is a significant global gastrointestinal issue, causing around 500,000 deaths annually in children under five (3). The World Health Organization (WHO) defines diarrhea as the passage of three or more loose or watery stools within a 24-h period (4). The disruption of intestinal microflora is a hallmark of diarrhea and can be triggered by various factors, including antibiotic use, infectious agents, and poor nutrition (5). Diarrhea can cause dehydration and electrolyte imbalances in children and adults, leading to serious consequences such as growth stunting in children, malnutrition, and recurrent enteric infections (6). This impairment is linked to a heightened risk of mortality (3, 7).

Also, constipation, characterized by infrequent and painful bowel movements, abdominal discomfort, and fecal incontinence, poses a major challenge in pediatric and adults healthcare worldwide. The prevalence of this condition is estimated to be between 0.7 and 29.6% worldwide (8). It is a common concern among both children and adults, often causing significant physical discomfort in affected individuals, along with psychological impacts (9). Constipation arises from a combination of factors, including genetic predisposition, disrupted intestinal motility, low dietary fiber and fluid intake, insufficient physical activity, and a diminished urge to defecate (10). Given these impacts of diarrhea and constipation on health and well-being, finding effective interventions is crucial. This is where probiotics come into play, offering a promising approach to managing and alleviating these gastrointestinal issues.

Probiotics have gained widespread recognition for their role in promoting gut health, particularly in preventing and managing gastrointestinal disorders like diarrhea and constipation. Their effectiveness is primarily linked to their ability to restore microbial balance, enhance gut barrier function, and modulate immune responses (11). Probiotics, defined by the World Health Organization as “live microorganisms that, when consumed in adequate amounts, confer health benefits to the host,” have demonstrated efficacy in managing gastrointestinal disorders such as diarrhea and constipation (12, 13). Probiotics exert their effects through a variety of mechanisms, including competitive inhibition of pathogenic bacteria, enhancement of mucosal barrier integrity, modulation of local and systemic immune responses, and production of antimicrobial compounds such as bacteriocins and short-chain fatty acids (14). The therapeutic efficacy of probiotics is strain-dependent. *Lactobacillus rhamnosus* GG enhances intestinal barrier function and stimulates the production of anti-inflammatory cytokines, while Saccharomyces boulardii has been shown to inhibit pathogen adhesion and increase enzyme activity that aids in nutrient absorption (15).

Numerous meta-analyses have evaluated the therapeutic effects of probiotics on diarrhea and constipation in the pediatric population (7, 9, 16–18). However, their findings remain inconsistent (9, 19–21), likely due to differences in statistical approaches and heterogeneity in study designs. To address these discrepancies, we applied a uniform statistical methodology to synthesize the evidence and provide a more definitive assessment of the effects of probiotic supplementation on diarrhea- and constipation-related outcomes in children.

## Methods

The present meta-analysis was conducted in accordance with the PRISMA guidelines (22). The protocol for this study has been documented in the International Prospective Register of Systematic Reviews (PROSPERO).

### Search strategy

A comprehensive systematic search was conducted on scientific databases, including PubMed, Scopus, EMBASE, Web of Science, and Google Scholar, covering the period from inception until December 2024. The search strategy was developed using a combination of MeSH terms and keywords. (“Probiotics” OR “Probiotics” [tiab] OR “probiotic” [tiab] OR “lactobacillus” OR “lactobacillus” [tiab] OR “Bifidobacterium” [tiab]) AND (“stool consistency” OR “stool frequency” [tiab] OR “diarrhea” [tiab] OR “constipation” [tiab]) AND (“pediatric populations” [tiab] OR “children” [tiab]) AND (“systematic review” [tiab] OR “meta-analysis” [tiab]).

### Inclusion and exclusion criteria

The PICO criteria for this umbrella meta-analysis were defined as follows: Population/Patients (P: both individuals under 18 years old receiving probiotic treatment); Intervention (I: administration of probiotics); Comparison (C: a control or placebo group); and Outcome (O: prevention of diarrhea, diarrhea incidence, duration of diarrhea, constipation, stool frequency and stool consistency). This umbrella review incorporated systematic reviews and meta-analysis that examined the impact of probiotic supplementation on diarrhea and constipation, specifically those that provided effect sizes (ESs) along with their respective confidence intervals (CIs). Conversely, the review excluded studies of an *in vitro*, *in vivo*, or ex vivo nature, as well as case reports, observational studies, quasi-experimental studies, and controlled clinical trials. Furthermore, the search was restricted to articles published in the English language.

### Methodological quality assessment

The methodological quality of the included articles was evaluated using the A Measurement Tool to Assess Systematic Reviews (AMSTAR) 2 questionnaire, which was administered by two independent researchers (23). The AMSTAR2 checklist is classified into four distinct quality categories: “critically low quality,” “low quality,” “moderate quality,” and “high quality.”

### Study selection and data extraction

Two independent reviewers, conducted a screening of the articles in accordance with the established eligibility criteria. Initially, the titles and abstracts of the articles were evaluated. Subsequently, the full texts of the remained articles were evaluated to determine their eligibility for inclusion in the current umbrella meta-analysis. Any discrepancies were discussed. The extracted data encompassed the outcomes, specifically ESs and CIs, along with details such as the name of the first author, year of publication, the geographical location, number of included studies in each meta-analysis, total sample sizes, and the outcome.

### Data synthesis and statistical analysis

The pooled ES and its associated 95% CI were estimated using random-effects models implemented via the restricted maximum likelihood (REML) approach (24). The Cochran-Q test and the I^2^ index were utilized to evaluate the heterogeneity within the meta-analysis. A significant level of heterogeneity in the data was established when I^2^ exceeded 50% or when the Cochran-Q test yielded a significant result (*p* < 0.10) (24). Subgroup analysis using predetermined variables—type of ES (WMD or SMD), health status, and single or multi-strain probiotics—helped identify potential sources of heterogeneity. A sensitivity analysis was performed to evaluate the impact of excluding a specific study on the overall ES. For outcomes that included a minimum of 10 studies, both Egger’s and Begg’s tests were applied, alongside a visual assessment of funnel plots, to explore the presence of small study effects (25–27). All statistical analyses were conducted using STATA version 16.0 (Stata Corporation, College Station, TX, US). A *p*-value of less than 0.05 was deemed significant.

## Results

### Study selection

**Figure 1:**
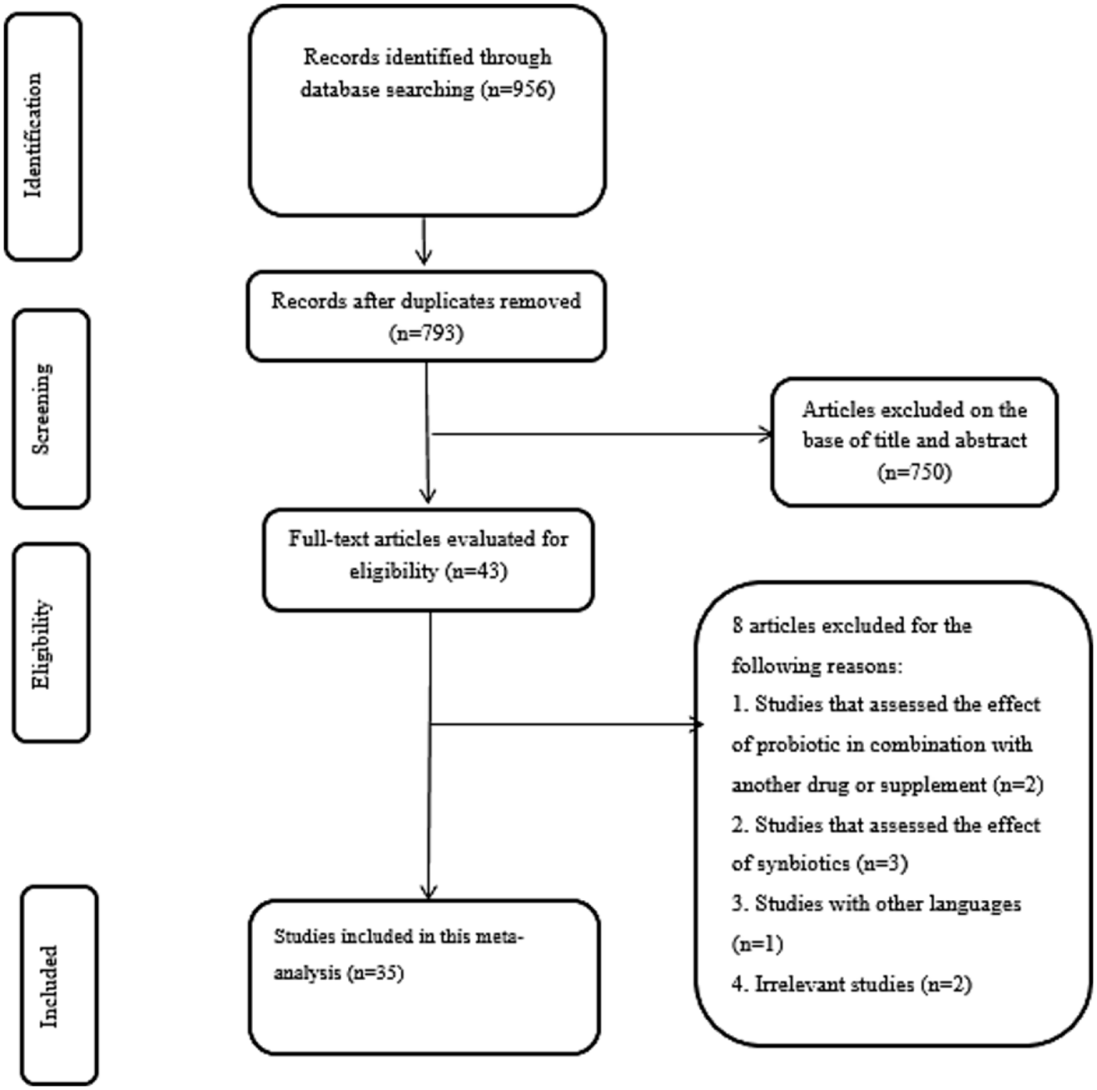
PRISMA flow diagram.

According to systematic search on above mentioned databases, 956 records were identified. Then, 163 duplicates were removed to screen the title and abstract of remained studies thoroughly. Afterward, 793 records were excluded and 43 studies were evaluated using full-text. Finally, eight studies were excluded by reason: studies that have used probiotics in combination with other compounds (*n* = 2), studies that assessed the effect of synbiotics (*n* = 3), studies with other languages (*n* = 1), and irrelevant studies (*n* = 2). In the end, a total of 35 studies met our specified inclusion criteria. A summary of the study selection process is provided in Figure 1.

### Study characteristics

In the present systematic review, a total of 35 systematic reviews were included (Table 1). All these studies were published between 2002 and 2024. The following are the number of meta-analyses for the outcomes across included studies: Diarrhea (prevention-RR): *n* = 4, Diarrhea (prevention-OR): *n* = 1, Diarrhea (Incidence-OR): *n* = 6, Diarrhea (Incidence-RR): *n* = 9, Diarrhea (Treatment-SMD): *n* = 3, Diarrhea duration: *n* = 19, Constipation: *n* = 3, and, Stool frequency (WMD): *n* = 3. The age range of included children and adults was <18 years old. The most used probiotics were *Lacticaseibacillus. acidophilus, Lacticaseibacillus. reuteri, Lacticaseibacillus. casei, Lacticaseibacillus. Bulgaricus, Streptococcus thermophilus, S. boulardii, Bifidobacterium, B. longum* and mix of probiotics (*Bifidobacterium, Lacticaseibacillus*, and *Streptococcus*).

**Table 1:** Study characteristics of included studies.

References	Location	No. of studies in meta-analysis	No. of participants in meta-analysis	Age (year)	Intervention	Health condition	Outcomes
Higuchi et al. (54)	Japan	7	878	<18	Probiotic	Acute gastroenteritis	Diarrhea incidence (↓ RR)
Higuchi et al. (54)	Japan	14	1761	<18	Probiotic	Acute gastroenteritis	↓ Diarrhea duration
Liang et al. (55)	China	4	1,465	<1	Probiotic	Infant	Diarrhea incidence (RR) (NS)
Fu et al. (56)	China	8	1,051	<7	*S. boulardii*	Children with diarrhea	↓ Diarrhea duration
Cheng et al. (57)	China	6	698	<12	*L. acidophilus*	Acute gastroenteritis	Diarrhea duration (NS)
Cheng et al. (57)	China	9	1765	<12	Mix	Acute gastroenteritis	↓ Diarrhea duration
Huang et al. (31)	China	6	668	<6	*L. reuteri, L. casei, S. boulardii, L. rhamnosus*	Children with diarrhea	Diarrhea incidence (↑ OR)
Huang et al. (31)	China	10	2,223	<6	*L. reuteri, L. rhamnosus, L. acidophilus, S. boulardii*, Mix	Children with diarrhea	↓ Diarrhea duration
Wu et al. (58)	China	12	1907	<5	*L. rhamnosus, L. acidophilus L. sporogenes, L. reuteri, S. boulardii*, Mix	Acute diarrhea	↓ Diarrhea duration
Szajewska et al. (59)	Poland	23	3,450	<15	*S. boulardii*	Acute gastroenteritis	↓ Diarrhea duration
Li et al. (60)	China	4	517	<3	*L. rhamnosus*	Acute pediatric diarrhea (>3 days)	Diarrhea incidence (↓ OR)
Li et al. (60)	China	3	475	<3	*L. rhamnosus*	Acute pediatric diarrhea (>4 days)	Diarrhea incidence (↓ OR)
Li et al. (60)	China	7	973	<5	*L. rhamnosus*	Acute pediatric diarrhea	↓ Diarrhea duration
Yang et al.(17)	China	12	925	15.86	Mix	Children with acute diarrhea	Diarrhea incidence (↓ RR)
Fang et al. (61)	China	3	358	<18	Lactobacillus	*Helicobacter pylori* patients	Diarrhea incidence (↓ RR)
Szajewska et al. (62)	Poland	15	3,820	<18	*L. rhamnosus*	Acute gastroenteritis	↓ Diarrhea duration
Patro-Goła et al. (63)	Poland	4	220	<5	*L. reuteri*	Acute gastroenteritis	↓ Diarrhea duration
Patro-Goł et al. (63)	Poland	2	160	<5	*L. reuteri*	Acute gastroenteritis with diarrhea	Stool frequency (NS)
Harris et al. (16)	Australia	11	835	<18	*L. reuteri*, Mix	Patients with functional constipation	Constipation (↑ RR)
Harris et al. (16)	Australia	14	965	<18	*L. reuteri*, Mix	Patients with functional constipation	Stool frequency (NS)
Ianiro et al. (64)	Italy	6	919	3.82	*Bacillus clausii*	Acute diarrhea	↓ Diarrhea duration
Ianiro et al. (64)	Italy	4	689	4.02	*Bacillus clausii*	Acute diarrhea	↓ Stool frequency
Jin et al. (21)	China	4	382	<18	*L. casei, L. rhamnosus, Bifidobacterium*	Functional constipated children	Constipation (RR) (NS)
Xu et al. (28)	China	21	7,225	<12	Bifidobacterium	Pediatric antibiotic-associated diarrhea	Prevention of diarrhea (↓ OR), Incidence diarrhea (↓ OR)
Huang et al. (9)	China	3	267	5.62	Mix	Constipated children	Stool consistency (NS)
Lau et al. (65)	US	4	888	<18	Mix	Inpatients and outpatients	Diarrhea incidence (↓ RR)
Urbanska et al. (66)	Poland	3	256	<18	*L. reuteri*	Diarrhoeal diseases	↓ Diarrhea duration
Szajewska et al. (67)	Poland	5	445	<18	*L. rhamnosus*	Antibiotic-associated diarrhea	Prevention of diarrhea (↓ RR)
Ahmadi et al. (68)	Iran	17	1,149	<6	*L. rhamnosus, L. casei, L. acidophilus, L. reuteri, S. boulardii*	Acute rotavirus diarrhea	↓ Diarrhea duration
Wanke et al. (69)	Poland	3	1,043	<18	*L. rhamnosus*	Healthcare-associated diarrhea	Prevention of diarrhea (↓ RR)
Li et al. (29)	China	3	217	<18	Mix	*Helicobacter pylori* patients	Diarrhea incidence (↓ OR)
Li et al. (29)	China	2	151	<18	Mix	*Helicobacter pylori* patients	Constipation (OR) (NS)
Szajewska et al. (70)	Poland	11	2,444	<6	Lactobacillus	Acute gastroenteritis	↓ Diarrhea duration
Videlock et al. (71)	US	10	1,246	<18	Probiotics	Antibiotic-associated diarrhea	Diarrhea incidence (↓ RR)
Salari et al. (72)	Iran	19	3,787	<18	*L. acidophilus, L. rhamnosus, L. paracasei, L. casei, S. boulardii, S. thermophilus*, Mix	Acute diarrhea	↓ Diarrhea duration
Szajewska et al. (73)	Poland	2	823	<18	*L. rhamnosus*	Healthcare-associated diarrhea	Diarrhea incidence (↓ RR)
Kale-Pradhan et al. (74)	US	4	585	<18	Lactobacillus	Antibiotic-associated diarrhea	Prevention of diarrhea (RR) (NS)
Szajewska et al. (75)	Poland	4	1,305	<18	*S. boulardii*	*Helicobacter pylori* patients	Diarrhea incidence (↓ RR)
Chmielewska et al. (76)	Poland	2	106	<18	*L. reuteri*	Acute infectious diarrhea	↓ Diarrhea duration
Szajews et al. (77)	Poland	7	876	<18	Lactobacillus	Acute infectious diarrhea	↓ Diarrhea duration
Szajewsk et al. (78)	Poland	4	473	<18	*S. boulardii*	Acute infectious diarrhea	↓ Diarrhea duration
Johnston et al.(19)		6	707	<18	*Lactobacillus, S. boulardii*	Antibiotic-associated diarrhea	Diarrhea incidence (↓ RR)
D’Souza et al. (50)	UK	9	623	<18	*Lactobacillus, S. boulardii*, Mix	Antibiotic associated diarrhea	Diarrhea incidence (↓ OR)
Huang et al. (18)	US	25	1917	<5	Mix	Acute diarrhea	↓ Diarrhea duration

### Risk of bias assessment

The risk of bias for included studies was assessed using AMSTAR questionnaire. Detailed results are presented in Supplementary Table 1.

### Probiotics supplementation on prevention of diarrhea

**Figure 2:**
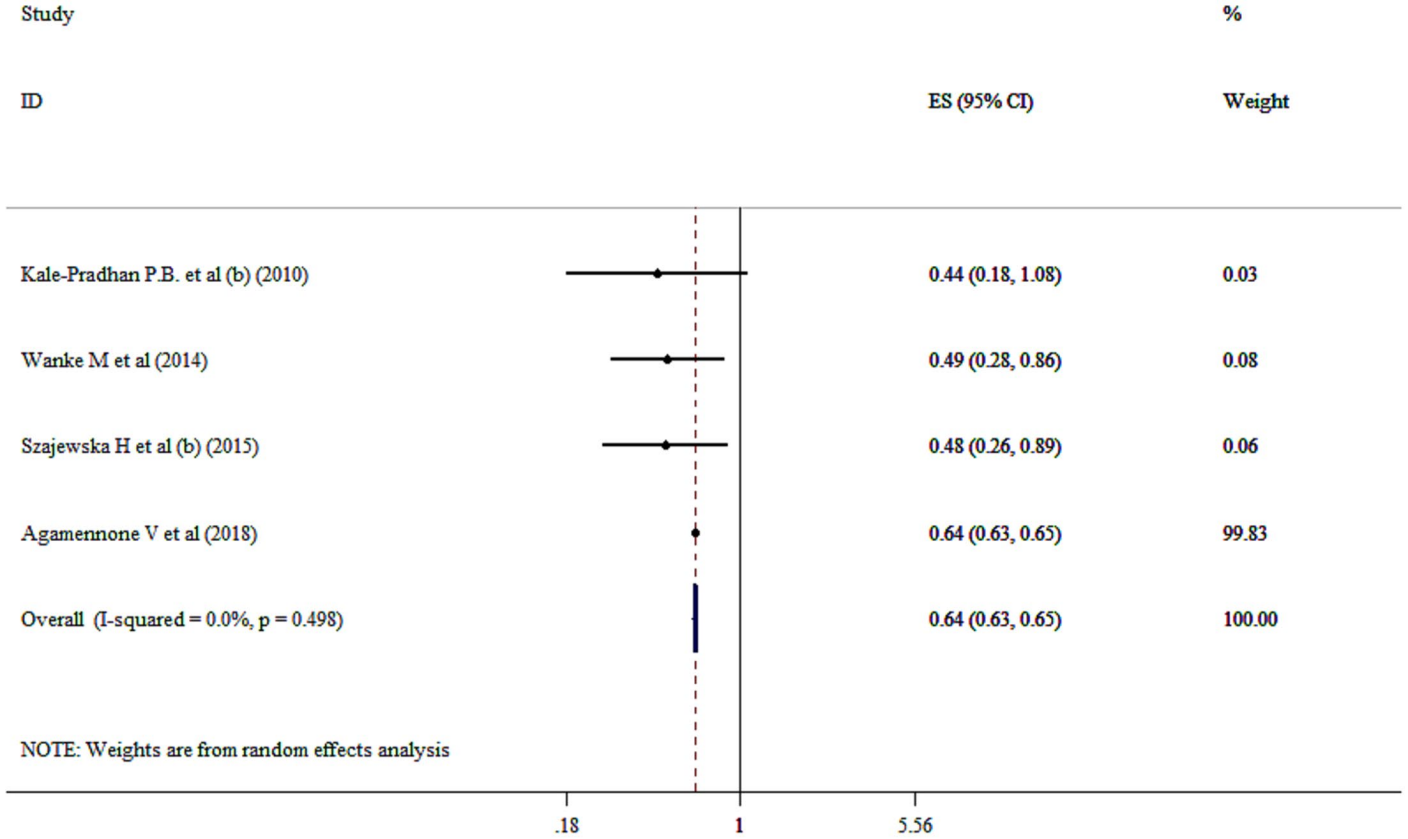
Mean difference and 95% CIs presented in forest plot of the studies on the effects of probiotics on prevention of diarrhea.

The utilized random effect model revealed that probiotics significantly reduced the relative risk of diarrhea by 36% compared to the control group (RR = 0.64; 95% CI: 0.63, 0.65, *p* < 0.001) without heterogeneity (*I*^2^ = 0.0%, *p* = 0.498) (Figure 2). This finding is highlighting potential of probiotics as an effective preventive strategy. However, one studies which have reported odds ratio (OR) for preventing diarrhea, were included in our systematic review (RR = 0.34; 95% CI: 0.28, 0.41, *p* < 0.05) (28). Moreover, sensitivity analysis demonstrated that no study could affect the pooled effect size. Furthermore, no evidence for publication bias based on Begg’s (*p* = 0.999).

### Probiotics supplementation on incidence of diarrhea (OR)

**Figure 3:**
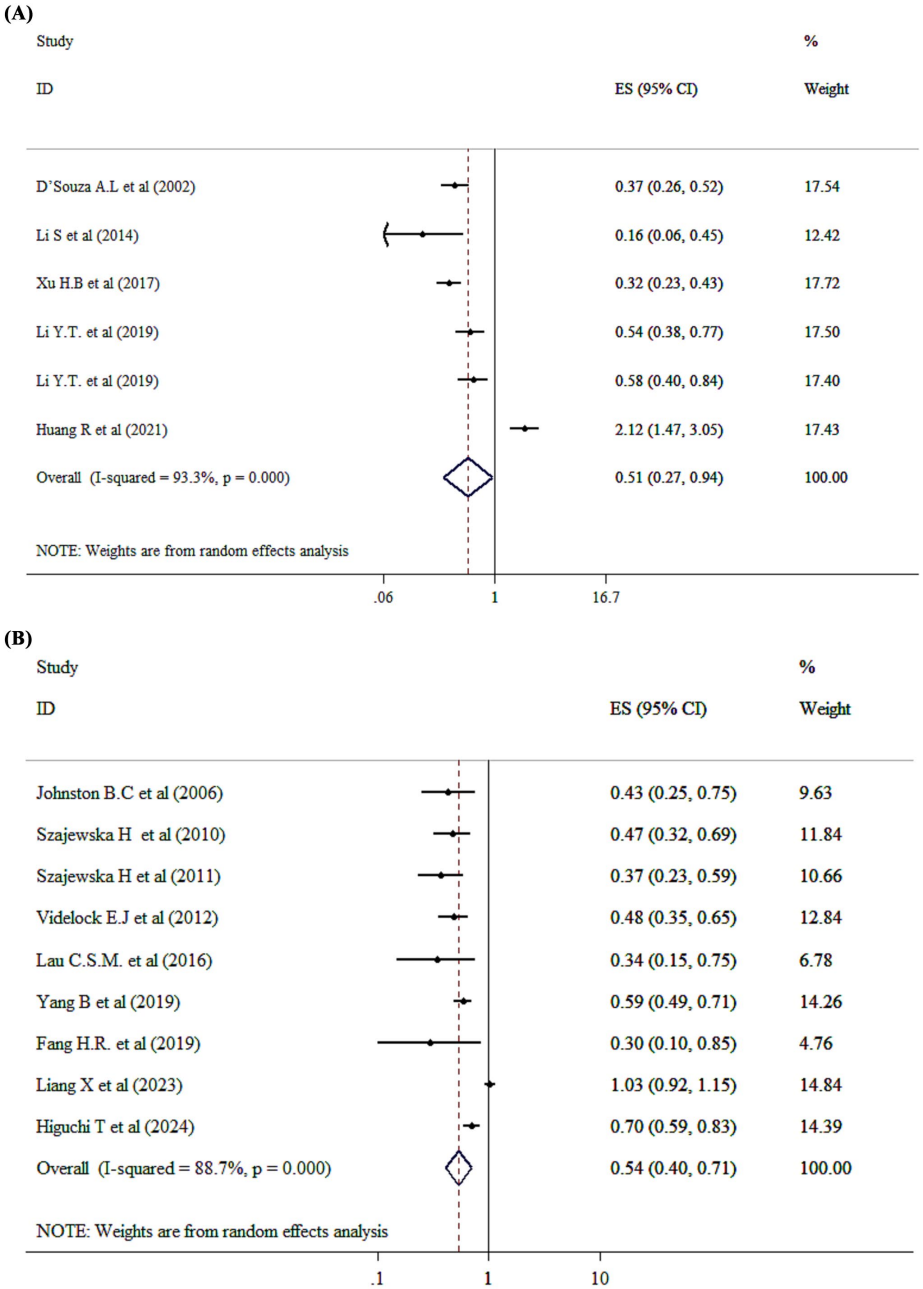
Mean difference and 95% CIs presented in forest plot of the studies on the effects of probiotics on incidence diarrhea based on OR **(A)**, and RR **(B)** analysis.

The pooled effect size revealed that probiotic intervention significantly reduced the odds of diarrhea incidence by 49% compared to the control group (OR = 0.51; 95% CI: 0.27, 0.94 *p* = 0.032; *I*^2^ = 93.3%, p-heterogeneity <0.001) (Figure 3A). Moreover, single-strain probiotics could exert beneficial effects in relation to reducing the OR of diarrhea incidence based on subgroup analysis (Supplementary Table 2). Furthermore, no single study effect was seen to affect the overall effect size of diarrhea incidence (OR). Furthermore, no evidence for publication bias was seen based on Begg’s (*p* = 0.904).

### Probiotics supplementation on incidence of diarrhea (RR)

The analysis of the impact of probiotics on diarrhea incidence (RR) involving 8,595 children. Probiotic administration has significantly decreased risk of diarrhea incidence by 46% compared to control group (RR = 0.54; 95% CI: 0.40, 0.71, *p* < 0.001) (Figure 3B). Besides, significant between-study heterogeneity has been detected (*I*^2^ = 88.7%, and *p* < 0.001). Additionally, both single-strain and multi-strain probiotics were associated with positive effects in reducing diarrhea incidence (RR) (Supplementary Table 2). Based on sensitivity analysis, no significant changes have been identified following removing one single study. Begg’s test has shown no significant publication bias (*p* = 0.09).

### Probiotics supplementation on duration of diarrhea

**Figure 4:**
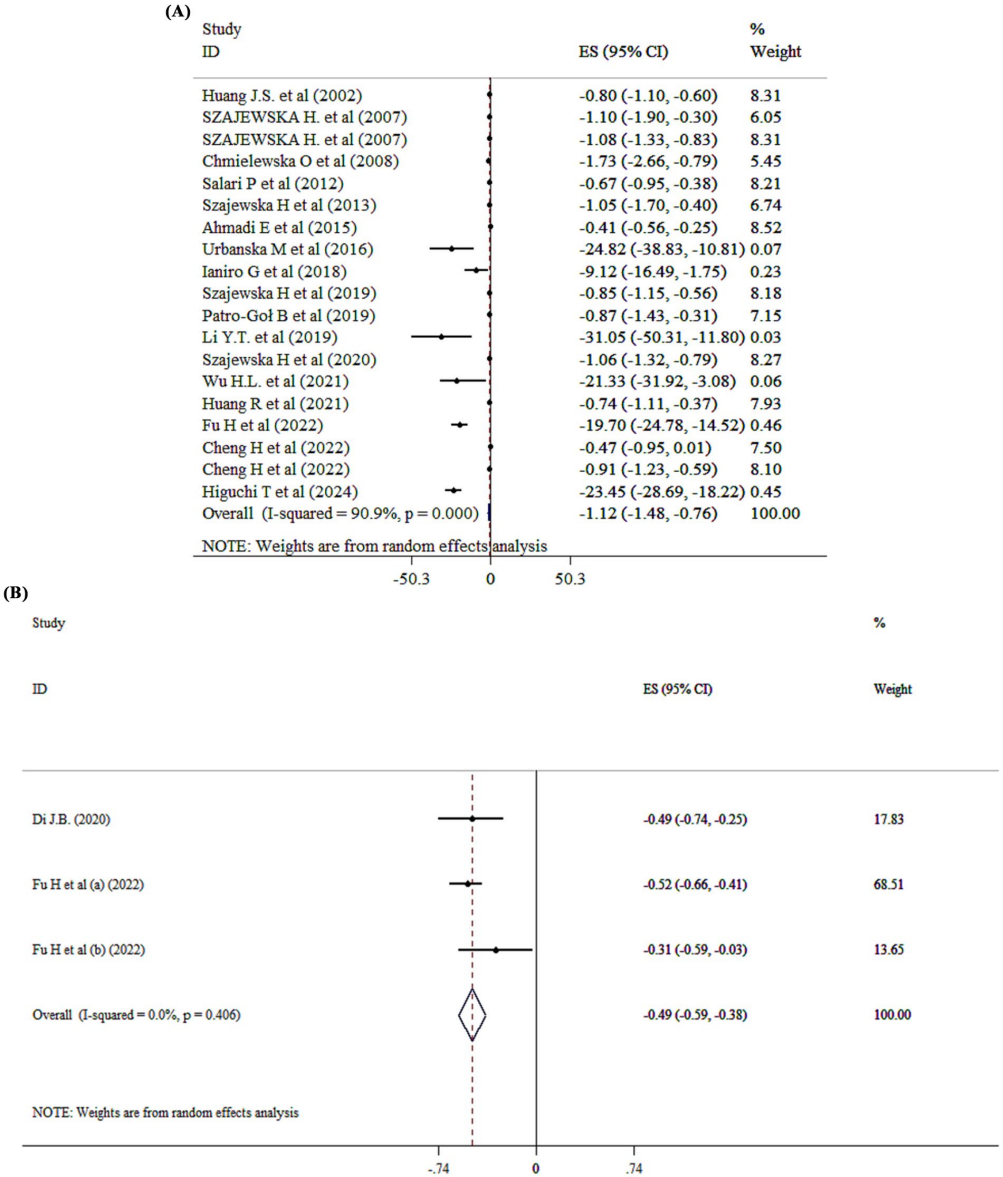
Mean difference and 95% CIs presented in forest plot of the studies on the effects of probiotics on duration of diarrhea **(A)**, and diarrhea treatment **(B)**.

The overall effect of probiotic supplementation on duration of diarrhea was analyzed across 18 studies with 19 ESs. The pooled analysis revealed a ES of −1.12 (95% CI: −1.48 to −0.76; *p* < 0.001; *I*^2^ = 90.9%, *p* < 0001) (Figure 4A). Subgroup analysis demonstrated that both single- and multi-strain probiotics are effective in reducing the period of diarrhea. Based on subgroup analysis both SMD analysis (SMD = −0.94; 95% CI: −1.32, −0.56, *p* < 0.001) and WMD analysis (WMD = −1.85; 95% CI: −2.83, −0.86, *p* < 0.001) demonstrated that probiotics are effective in reducing the period of diarrhea (Supplementary Table 2). The effects of probiotics on acute diarrhea and diarrheal diseases were stronger than in other subgroups (Supplementary Table 2). In addition, sensitivity analysis revealed that no significant change has been detected following removing one single study. Begg’s and Egger’s tests, and visual inspection of funnel plot pointed to significant publication bias (*p* < 0.005).

### Probiotics supplementation on diarrhea treatment

Probiotic administration significantly improved diarrhea treatment (SMD = −0.49; 95% CI: −0.59, −0.38, *p* < 0001) (Figure 4B).

### Probiotics supplementation on constipation

**Figure 5:**
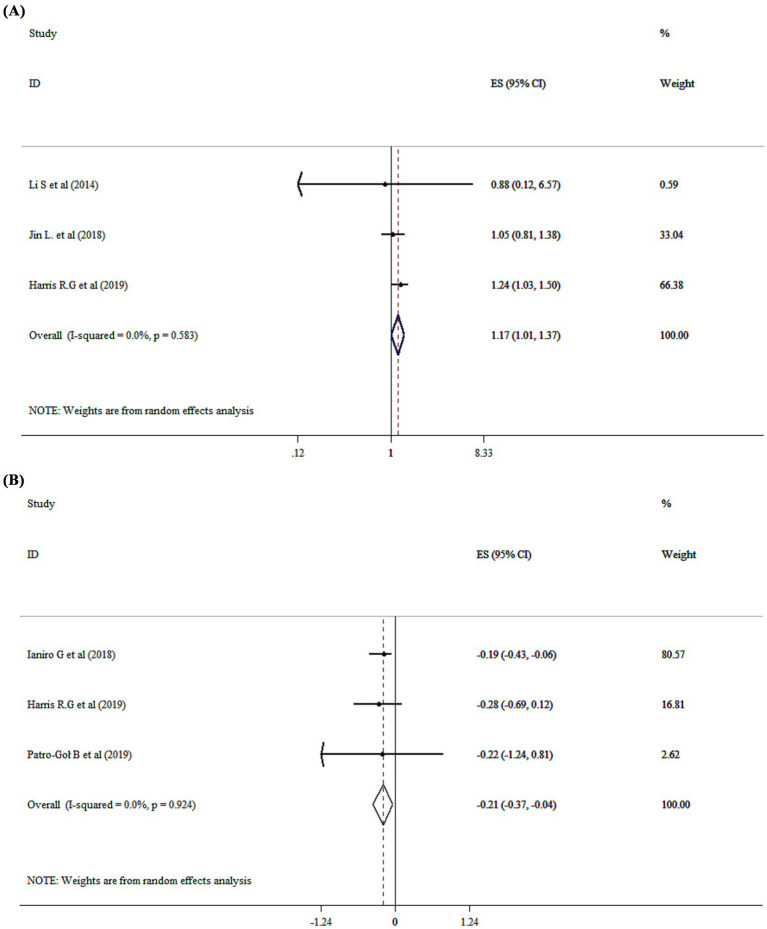
Mean difference and 95% CIs presented in forest plot of the studies on the effects of probiotics on constipation **(A)**, and stool frequency **(B)**.

The overall analysis revealed that probiotics significantly impact on constipation (OR = 1.17, 95% CI: 1.01 to 1.37; *p* = 0.043, *I*^2^ = 0.0%, *p* = 0.583) (Figure 5A). Three meta-analyses (16, 21, 29) demonstrated limited and inconsistent evidence supporting probiotic use for pediatric functional constipation. While some strains showed modest improvements in stool frequency, significant heterogeneity in study design, dosage, and outcome measures restricted definitive conclusions. These findings underscore the importance of strain- and condition-specific research before recommending probiotics for functional constipation in children.

### Probiotics supplementation on stool frequency and consistency

Probiotics significantly affected stool frequency with a WMD of −0.21 (95% CI: −0.37 to −0.04; *p* = 0.015; *I*^2^ = 0.0%, *p* = 0.924) (Figure 5B), but had no effect on stool consistency (WMD = −0.07; 95% CI: −0.21, 0.06, *P* ˃0.05).

## Discussion

The present umbrella of systematic reviews and meta-analysis attempted to summarize the available data evaluating the effect of probiotics on diarrhea and constipation outcomes among children. Accordingly, probiotic supplementation significantly reduced the RR values for diarrhea incidence and prevention in children compared to the control group. This outcome is consistent with a Cochrane review, which concluded that probiotics are effective in preventing antibiotic-associated diarrhea (AAD) in children, with specific strains showing increased effectiveness (30). Also, probiotic supplementation was associated with a reduced odds of diarrhea incidence in children specifically. In addition, probiotics were able to shorten the duration of diarrhea in study subjects. Focusing on pediatric populations, a meta-analysis demonstrated that the duration of diarrhea in children receiving probiotics was significantly shorter than in control groups, and the length of hospital stay was also reduced (31). All these findings point to beneficial effects of probiotics as an adjunctive approach in managing diarrhea across different age groups.

The types of diarrhea included across the analyzed studies varied and encompassed AAD, infectious diarrhea, and cases where the etiology was not explicitly stated. This heterogeneity may influence the pooled estimates, as the efficacy of probiotics is known to differ depending on the underlying cause (30). For example, probiotics such as *Lactobacillus rhamnosus* and *Saccharomyces boulardii* have demonstrated greater efficacy in managing infectious and antibiotic-associated diarrhea, respectively (32). Clinicians should consider probiotic strains with well-established efficacy, such as *Lactobacillus rhamnosus* and *Saccharomyces boulardii*, administered at dosages typically ranging from 10^9^ to 10^10^ colony-forming units (CFU) per day, consistent with doses used in clinical trials (33, 34). Accordingly, the variability in diarrhea type should be considered when interpreting the overall results, and future meta-analyses may benefit from stratifying outcomes based on etiology to enhance clinical relevance.

Based on the available evidence, some probiotic strains were more effective in improving gastrointestinal outcomes in children. *Lactobacillus rhamnosus* GG and Saccharomyces boulardii were consistently associated with significant reductions in the duration and frequency of acute diarrhea, particularly in cases of viral or antibiotic-associated etiology (33, 34). These findings are supported by several high-quality randomized controlled trials with low risk of bias. In contrast, the evidence for constipation was more variable, with *Bifidobacterium lactis* and *Lactobacillus casei rhamnosus* showing moderate effectiveness in improving stool frequency and consistency (35). However, the quality of evidence for constipation-related outcomes was generally lower, often limited by small sample sizes and heterogeneous outcome measures.

Moreover, probiotics resulted in decreased stool frequency in children. While, Dong et al., demonstrated improved defecation frequency following probiotic treatment in children (36). This discrepancy is justified by smaller number of included studies and lower sample size in Dong et al.’s study (36), which may affect the statistical power to detect significant changes. Also, this issue shed the light that microecological differences between children and adults may lead to variations in constipation-related outcomes.

Furthermore, odds of diarrhea incidence were significantly affected by single-strain probiotics. Whereas both single-strain and multi-strain probiotics effectively reduced diarrhea duration, as well as the risk of diarrhea incidence. It is worth noting that while single -strain probiotics demonstrated beneficial effects on diarrhea-related outcomes and stool frequency, they were also shown to increase stool consistency in constipated patients. Given this, caution is warranted in interpreting these findings. Accordingly, Schnadower et al. demonstrated that probiotics single-strain (*Lactobacillus rhamnosus*) was ineffective in the treatment of acute enteritis in children (37). Similarly, Szymański et al. reported no significant changes in relation to diarrhea symptoms following *Lactobacillus reuteri* administration (38). However, Zhang et al. highlighted that enhanced diversity of microbiota can improve constipation-related measures significantly. This underscores the effectiveness of multi-strain probiotics in addressing constipation (39). Previous studies have demonstrated strain-specific effects of probiotics in different health status (40–42). For example, in constipated children some probiotics have been known as discriminative species such as *Bacteroides* and *Bifidobacterium longum* species (43).

Probiotic administration resulted in significant improvements in reducing the duration and frequency of diarrhea in children. This effect was particularly significant in cases of acute viral diarrhea. The therapeutic efficacy of probiotics is also strain-dependent, with specific strains such as *Lactobacillus rhamnosus* and *Saccharomyces boulardii* showing the greatest benefit (44). Furthermore, while single-strain and multi-strain formulations have been used, current evidence suggests that clinical outcomes are more influenced by individual characteristics and strain viability than by the number of strains present (45). In addition, probiotic supplementation serves as an adjunct to standard diarrhea management protocols.

The relatively modest impact of probiotics on constipation observed in pediatric populations may be attributed to several factors. Constipation is a multifactorial disorder influenced by diet, hydration, physical activity, gut motility, and psychosocial elements, which probiotics alone may not fully address (35). Additionally, the heterogeneity of probiotic strains studied, differences in dosage, and variability in intervention duration complicate the interpretation of efficacy (46). The mechanisms by which probiotics might alleviate constipation—such as modulation of gut microbiota composition, enhancement of short-chain fatty acid production, and improvement of intestinal transit—may require longer treatment periods or higher doses than those employed in existing trials (47). Furthermore, outcome measures in constipation studies often vary widely, and subjective symptom reporting can affect the reliability of results. Finally, individual differences in baseline microbiota composition may influence response to probiotic supplementation, underscoring the need for personalized approaches in future research (48).

Several underlying mechanisms have been proposed through which probiotics influence gastrointestinal disorders such as diarrhea and constipation. Probiotics are known to modulate gut microbiota composition, helping to restore microbial balance. It has been shown that probiotics compete with pathogenic bacteria for adhesion sites which may affect the incidence of infectious diseases causing diarrhea and alleviating constipation (49). On the other hand, probiotics stimulate the intestinal immune cells and commensal microflora to regulate immune responses by activation of regulatory T cells (Tregs) (50, 51). Also, probiotics can improve the integrity of the intestinal barrier through strengthening tight junctions. This action prevents the adhesion of pathogenic bacteria to mucosal epithelial cells, and prevents translocation of harmful pathogens and toxins (49, 51). Moreover, probiotics were effective in alleviating inflammation state associated with diarrhea (52). It seems that each of these mechanisms may be possible through various strains. For example, *Saccharomyces boulardii* and *lactobacilli* modify immune pathways to remove the pathogens (50). *Lactobacilli* increases the production of immunoglobulins in the gut which contribute to production of interferons. It has been demonstrated that *Lactobacillus rhamnosus GG (LGG)* could produce antimicrobial substances to inhibits the growth of *Escherichia coli*, *streptococci*, and *Clostridium difficile* (50).

Although probiotics are generally considered safe for use in children, especially in healthy children, long-term safety data remain limited. Most clinical trials have focused on short-term administration during acute illness, and few studies have evaluated long-term or repeated use of probiotics. Rare adverse events, such as infections in immunocompromised individuals, highlight the need for cautious use in vulnerable populations (33, 34). Furthermore, the variability in probiotic formulations and lack of close regulatory oversight may lead to inconsistent safety profiles. Adherence to probiotic supplementation can also affect clinical efficacy, particularly in chronic conditions such as constipation where long-term administration may be necessary. Factors that influence adherence include: formulation palatability, dosing frequency, and caregiver education (53). Ensuring user-friendly formulations and clear instructions can improve adherence and optimize outcomes. Future research should prioritize long-term safety monitoring and explore strategies to increase adherence in pediatric populations.

As a strength, this study is a high-level research synthesis method that integrates findings from multiple meta-analyses in relation to effects of probiotics on diarrhea and constipation outcomes. So, this study provides more comprehensive overview of probiotics evidence, reliable conclusions and bias-minimized assessment. Moreover, this study included larger population to analyze the effects of probiotics in children. However, this study had some limitations too. First, several factors including: various type of preparation, purity, storing methods and cold chain principles (maintaining appropriate temperature conditions during storage and transportation to preserve the viability of live microorganisms) may result in different outcomes. Second, the number of studies on diarrhea caused by radiation, chemotherapy, HIV, and *Clostridium difficile*–associated diarrhea was insufficient to reach a confirm conclusion. Third, it is important to highlight changes in the intestinal flora, which were not reported in the included studies. Forth, included studies were heterogeneous in term of sex distribution.

## Conclusion

This umbrella of meta-analysis supports the potential role of probiotics in relation to diarrhea and constipation outcome in children. Probiotic supplementation contributed to a declined risk and odds of diarrhea incidence compared to control group. Additionally, they were able to shorten the duration of diarrhea and did not increase stool frequency and consistency too. Furthermore, subgroup analysis revealed that both single and multi-strain probiotics were able to reduce the diarrhea duration and the risk of diarrhea incidence. Therefore, further investigations on special types of the probiotics, their purity, and their combination with prebiotics may be much helpful.

## Data Availability

The datasets presented in this study can be found in online repositories. The names of the repository/repositories and accession number(s) can be found in the article/Supplementary material.
