# Supplementary material for: The effect of probiotics on the diarrhea and constipation outcomes in children: an umbrella review of systematic reviews and meta-analyses
Source: Front Nutr. 2025 Jul 18;12:1606264. doi: 10.3389/fnut.2025.1606264 (PMC12315117; doi:10.3389/fnut.2025.1606264)
Supplement: Supplementary file 1 [file Supplementary_File_1.DOCX]

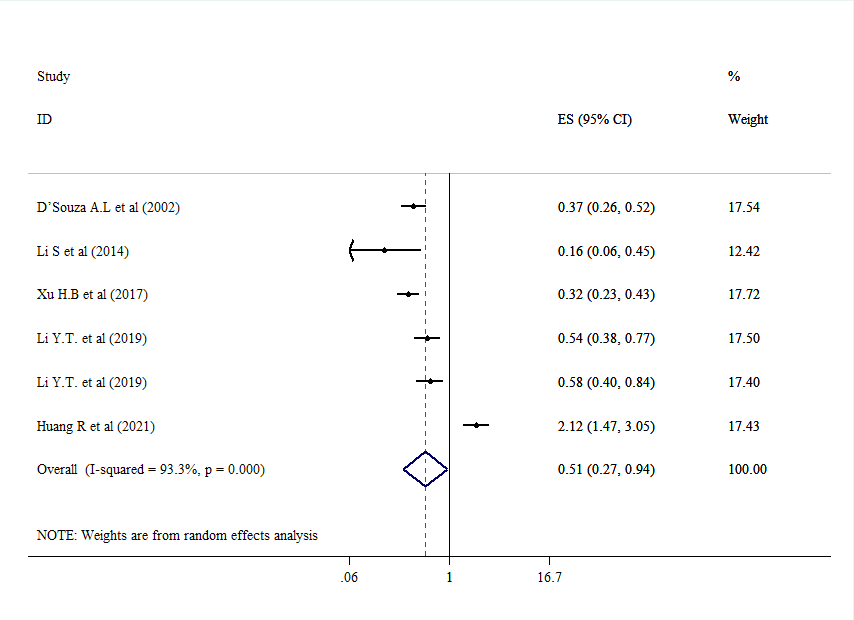
 **Figure S1.** Mean difference and 95% CIs presented in forest plot of the studies on the effects of probiotics on incidence diarrhea based on OR analysis.


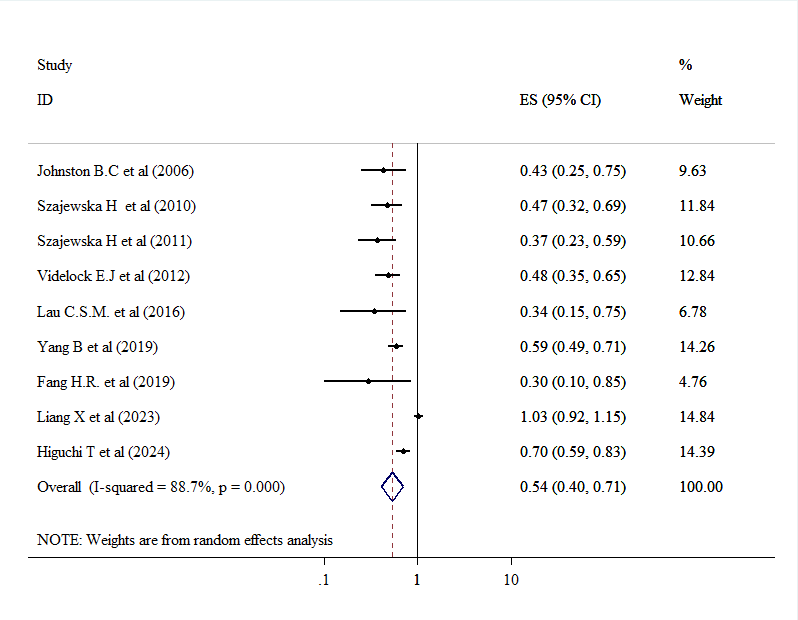
**Figure S2.** Mean difference and 95% CIs presented in forest plot of the studies on the effects of probiotics on incidence diarrhea based on RR analysis.


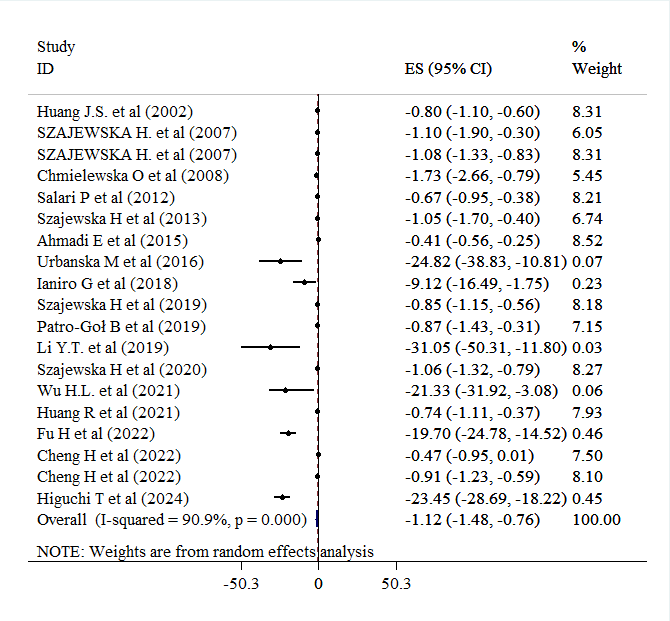


**Figure S3.** Mean difference and 95% CIs presented in forest plot of the studies on the effects of probiotics on duration of diarrhea.


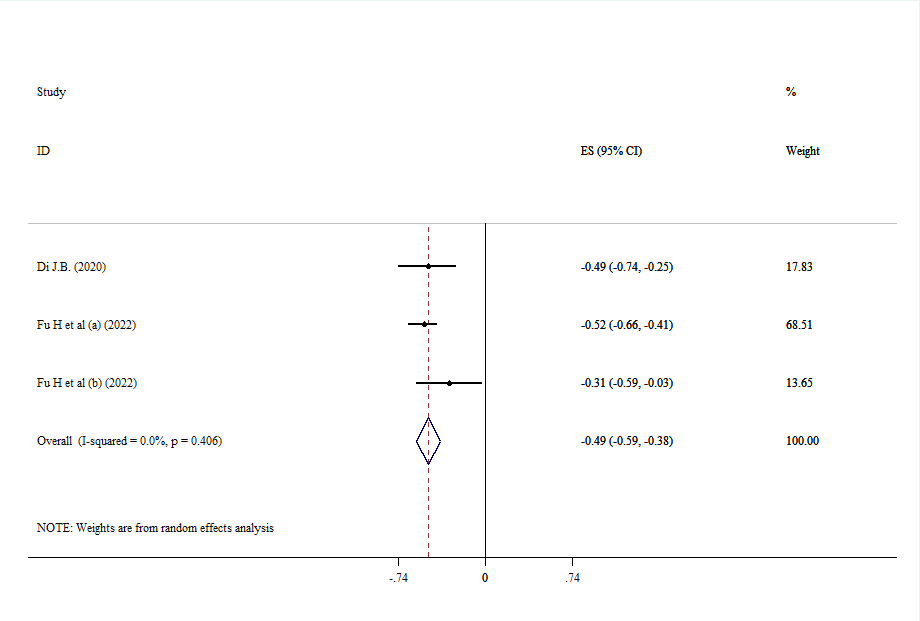
**Figure S4.** Mean difference and 95% CIs presented in forest plot of the studies on the effects of probiotics on diarrhea treatment.

**
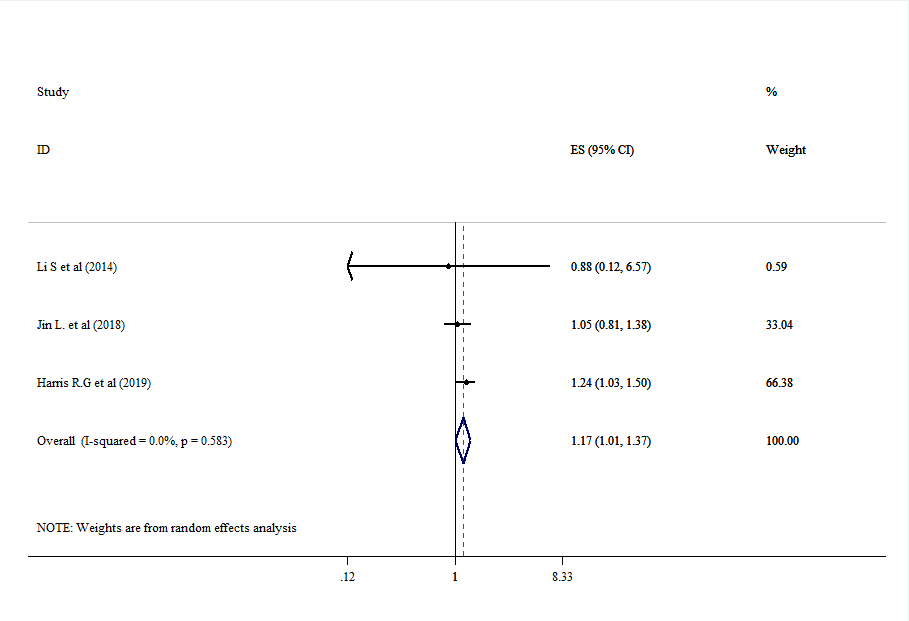
Figure S5.** Mean difference and 95% CIs presented in forest plot of the studies on the effects of probiotics on constipation.


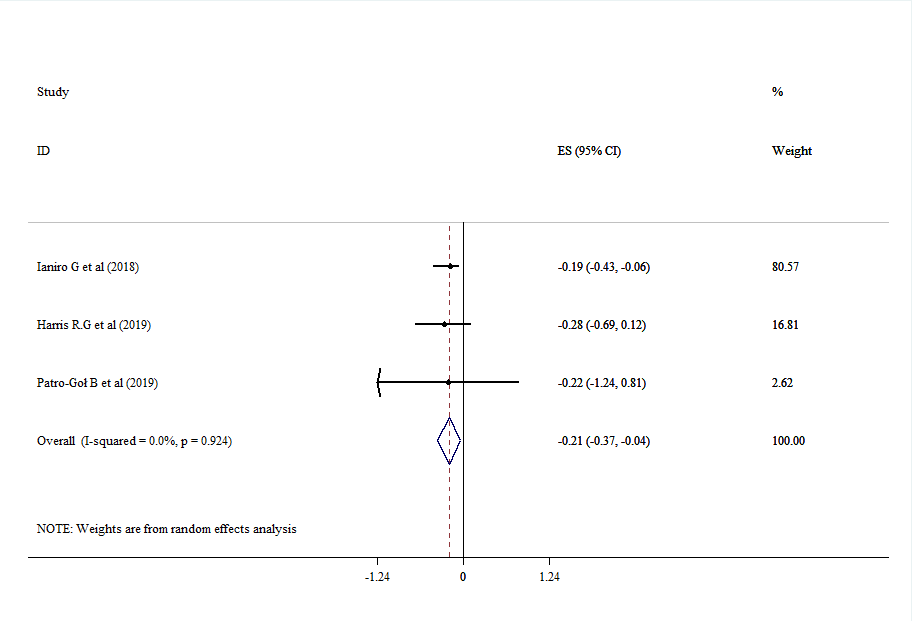


**Figure S6.** Mean difference and 95% CIs presented in forest plot of the studies on the effects of probiotics on stool frequency.

**Table S1**. Results of assess the methodological quality of meta-analysis.

| **Authors, Year** | **A priori design** | **Selection and data extraction** | **Literature search** | **Publication type** | **List of studies** | **Characteristics of the included studies** | **Assessed scientific quality** | **Scientific quality formulating conclusions** | **Methods used to combine the findings** | **Assessed** **publication bias** | **Conflict of interest stated** |
| --- | --- | --- | --- | --- | --- | --- | --- | --- | --- | --- | --- |
| Higuchi T et al, 2024 ^1^ | + | + | + | - | + | + | + | + | + | + | + |
| Liang X et al, 2023 ^2^ | + | + | + | - | + | + | + | + | + | + | + |
| Fu H et al, 2022 ^3^ | - | + | + | - | + | + | + | ? | + | + | + |
| Cheng H et al, 2022 ^4^ | + | + | + | - | + | + | + | + | + | + | + |
| Huang R et al, 2021 ^5^ | - | + | + | - | + | + | + | + | + | + | + |
| Wu H.L. et al, 2021 ^6^ | + | + | + | - | + | + | + | + | + | + | + |
| Szajewska H et al, 2020 ^7^ | + | + | + | - | + | + | + | + | + | + | + |
| Li Y.T. et al, 2019 ^8^ | - | + | + | - | + | + | + | + | + | + | + |
| Yang B et al, 2019 ^9^ | - | + | + | - | + | + | + | + | + | + | + |
| Fang H.R. et al, 2019 ^10^ | - | + | + | - | + | + | + | - | + | + | + |
| Szajewska H et al, 2019 ^11^ | + | + | + | - | + | + | + | + | + | + | + |
| Patro-GołaB et al, 2019 ^12^ | - | + | + | - | + | + | + | + | + | + | + |
| Harris R.G et al, 2019 ^13^ | + | + | + | - | + | + | + | + | + | + | + |
| Ianiro G et al, 2018 ^14^ | - | + | + | - | + | + | + | ? | + | + | + |
| Jin L. et al, 2018 ^15^ | - | + | + | - | + | + | + | - | + | + | + |
| Xu H.B et al, 2017 ^16^ | - | + | + | - | + | + | + | + | + | + | + |
| Huang R et al, 2017 ^17^ | - | + | + | - | + | + | + | - | + | + | + |
| Lau C.S.M. et al, 2016 ^18^ | - | + | + | - | + | + | + | - | + | + | + |
| Urbanska M et al, 2016 ^19^ | + | + | + | - | + | + | + | - | + | + | + |
| Szajewska H et al (b), 2015 ^20^ | - | + | + | - | + | + | + | + | + | + | + |
| Ahmadi E et al, 2015 ^21^ | - | + | + | - | + | + | + | - | + | + | + |
| Wanke M et al, 2014 ^22^ | - | + | + | - | + | + | + | + | + | + | + |
| Li S et al, 2014 ^23^ | - | + | + | - | + | + | + | + | + | + | + |
| Szajewska H et al, 2013 ^24^ | - | + | + | - | + | + | + | + | + | + | + |
| Videlock E.J et al, 2012 ^25^ | + | + | + | - | + | + | + | ? | + | + | + |
| Salari P et al, 2012 ^26^ | + | + | + | - | + | + | + | - | + | + | + |
| Szajewska H et al, 2011 ^27^ | + | + | + | - | + | + | + | - | + | + | + |
| Kale-Pradhan P.B. et al (b), 2010 ^28^ | - | + | + | - | + | + | + | - | + | + | + |
| Szajewska H et al, 2010 ^29^ | + | + | + | - | + | + | + | + | + | + | + |
| Chmielewska O et al, 2008 ^30^ | + | + | + | - | + | + | + | + | + | + | + |
| SZAJEWSKA H. et al, 2007 ^31^ | + | + | + | - | + | + | + | + | + | + | + |
| SZAJEWSKA H. et al, 2007 ^32^ | + | + | + | - | + | + | + | + | + | + | + |
| Johnston B.C et al, 2006 ^33^ | ? | + | + | - | + | + | + | - | + | + | + |
| D’Souza A.L et al, 2002 ^34^ | - | + | + | - | + | + | + | ? | + | + | + |
| Huang J.S. et al, 2002 ^35^ | - | + | + | - | + | + | + | - | + | + | + |

The result of assess the methodological quality using AMSTAR: each item for included studies (+: means yes; -: means no; ?: can't answer;).

**1.** Higuchi T, Furuichi M, Maeda N, Tsugawa T, Ito K. Effects of probiotics in children with acute gastroenteritis: A systematic review and meta-analysis focusing on probiotics utilized in Japan. *Journal of infection and chemotherapy : official journal of the Japan Society of Chemotherapy.* 2024;30:337-342.

**2.** Liang X, Li Y, Zhao Z, Ding R, Sun J, Chi C. Safety and efficacy of adding postbiotics in infant formula: a systematic review and meta-analysis. *Pediatric research.* 2024;95:43-51.

**3.** Fu H, Li J, Xu X, Xia C, Pan Y. Effectiveness and Safety of Saccharomyces Boulardii for the Treatment of Acute Gastroenteritis in the Pediatric Population: A Systematic Review and Meta-Analysis of Randomized Controlled Trials. *Computational and mathematical methods in medicine.* 2022;2022:6234858.

**4.** Cheng H, Ma Y, Liu X, Tian C, Zhong X, Zhao L. A Systematic Review and Meta-Analysis: Lactobacillus acidophilus for Treating Acute Gastroenteritis in Children. *Nutrients.* 2022;14.

**5.** Huang R, Xing HY, Liu HJ, Chen ZF, Tang BB. Efficacy of probiotics in the treatment of acute diarrhea in children: a systematic review and meta-analysis of clinical trials. *Translational pediatrics.* 2021;10:3248-3260.

**6.** Wu HL, Zhan X. Systematic review with meta-analysis: Probiotics for treating acute diarrhoea in children with dehydration. *Journal of paediatrics and child health.* 2021;57:431-439.

**7.** Szajewska H, Kołodziej M, Zalewski BM. Systematic review with meta-analysis: Saccharomyces boulardii for treating acute gastroenteritis in children—a 2020 update. *Alimentary Pharmacology and Therapeutics.* 2020;51:678-688.

**8.** Li YT, Xu H, Ye JZ, et al. Efficacy of Lactobacillus rhamnosus GG in treatment of acute pediatric diarrhea: A systematic review with meta-analysis. *World journal of gastroenterology.* 2019;25:4999-5016.

**9.** Yang B, Lu P, Li MX, et al. A meta-analysis of the effects of probiotics and synbiotics in children with acute diarrhea. *Medicine.* 2019;98:e16618.

**10.** Fang HR, Zhang GQ, Cheng JY, Li ZY. Efficacy of Lactobacillus-supplemented triple therapy for Helicobacter pylori infection in children: a meta-analysis of randomized controlled trials. *European journal of pediatrics.* 2019;178:7-16.

**11.** Szajewska H, Kołodziej M, Gieruszczak-Białek D, Skórka A, Ruszczyński M, Shamir R. Systematic review with meta-analysis: Lactobacillus rhamnosus GG for treating acute gastroenteritis in children – a 2019 update. *Alimentary Pharmacology and Therapeutics.* 2019;49:1376-1384.

**12.** Patro-Gołąb B, Szajewska H. Systematic Review with Meta-Analysis: Lactobacillus reuteri DSM 17938 for Treating Acute Gastroenteritis in Children. An Update. *Nutrients.* 2019;11.

**13.** Harris RG, Neale EP, Ferreira I. When poorly conducted systematic reviews and meta-analyses can mislead: a critical appraisal and update of systematic reviews and meta-analyses examining the effects of probiotics in the treatment of functional constipation in children. *The American journal of clinical nutrition.* 2019;110:177-195.

**14.** Ianiro G, Rizzatti G, Plomer M, et al. Bacillus clausii for the Treatment of Acute Diarrhea in Children: A Systematic Review and Meta-Analysis of Randomized Controlled Trials. *Nutrients.* 2018;10.

**15.** Jin L, Deng L, Wu W, Wang Z, Shao W, Liu J. Systematic review and meta-analysis of the effect of probiotic supplementation on functional constipation in children. *Medicine.* 2018;97:e12174.

**16.** Xu HB, Jiang RH, Sheng HB. Meta-analysis of the effects of Bifidobacterium preparations for the prevention and treatment of pediatric antibiotic-associated diarrhea in China. *Complementary therapies in medicine.* 2017;33:105-113.

**17.** Huang R, Hu J. Positive Effect of Probiotics on Constipation in Children: A Systematic Review and Meta-Analysis of Six Randomized Controlled Trials. *Frontiers in cellular and infection microbiology.* 2017;7:153.

**18.** Lau CSM, Chamberlain RS. Probiotics are effective at preventing Clostridium difficile-associated diarrhea: A systematic review and meta-analysis. *International journal of general medicine.* 2016;9:27-37.

**19.** Urbańska M, Gieruszczak-Białek D, Szajewska H. Systematic review with meta-analysis: Lactobacillus reuteri DSM 17938 for diarrhoeal diseases in children. *Alimentary pharmacology & therapeutics.* 2016;43:1025-1034.

**20.** Szajewska H, Kołodziej M. Systematic review with meta-analysis: Lactobacillus rhamnosus GG in the prevention of antibiotic-associated diarrhoea in children and adults. *Alimentary pharmacology & therapeutics.* 2015;42:1149-1157.

**21.** Ahmadi E, Alizadeh-Navaei R, Rezai MS. Efficacy of probiotic use in acute rotavirus diarrhea in children: A systematic review and meta-analysis. *Caspian journal of internal medicine.* 2015;6:187-195.

**22.** Wanke M, Szajewska H. Probiotics for preventing healthcare-associated diarrhea in children: A meta-analysis of randomized controlled trials. *Pediatria Polska.* 2014;89:8-16.

**23.** Li S, Huang XL, Sui JZ, et al. Meta-analysis of randomized controlled trials on the efficacy of probiotics in Helicobacter pylori eradication therapy in children. *European journal of pediatrics.* 2014;173:153-161.

**24.** Szajewska H, Skórka A, Ruszczyński M, Gieruszczak-Białek D. Meta-analysis: Lactobacillus GG for treating acute gastroenteritis in children--updated analysis of randomised controlled trials. *Alimentary pharmacology & therapeutics.* 2013;38:467-476.

**25.** Videlock EJ, Cremonini F. Meta-analysis: probiotics in antibiotic-associated diarrhoea. *Alimentary pharmacology & therapeutics.* 2012;35:1355-1369.

**26.** Salari P, Nikfar S, Abdollahi M. A meta-analysis and systematic review on the effect of probiotics in acute diarrhea. *Inflammation & allergy drug targets.* 2012;11:3-14.

**27.** Szajewska H, Wanke M, Patro B. Meta-analysis: the effects of Lactobacillus rhamnosus GG supplementation for the prevention of healthcare-associated diarrhoea in children. *Alimentary pharmacology & therapeutics.* 2011;34:1079-1087.

**28.** Kale-Pradhan PB, Jassal HK, Wilhelm SM. Role of Lactobacillus in the prevention of antibiotic-associated diarrhea: a meta-analysis. *Pharmacotherapy.* 2010;30:119-126.

**29.** Szajewska H, Horvath A, Piwowarczyk A. Meta-analysis: the effects of Saccharomyces boulardii supplementation on Helicobacter pylori eradication rates and side effects during treatment. *Alimentary pharmacology & therapeutics.* 2010;32:1069-1079.

**30.** Chmielewska A, Ruszczyński M, Szajewska H. Lactobacillus reuteri strain ATCC 55730 for the treatment of acute infectious diarrhoea in children: A meta-analysis of randomized controlled trials. *Pediatria Wspolczesna.* 2008;10:32-36.

**31.** Szajewska H, Skórka A, Ruszczyński M, Gieruszczak-Białek D. Meta-analysis: Lactobacillus GG for treating acute diarrhoea in children. *Alimentary pharmacology & therapeutics.* 2007;25:871-881.

**32.** Szajewska H, Skórka A, Dylag M. Meta-analysis: Saccharomyces boulardii for treating acute diarrhoea in children. *Alimentary pharmacology & therapeutics.* 2007;25:257-264.

**33.** Johnston BC, Supina AL, Vohra S. Probiotics for pediatric antibiotic-associated diarrhea: a meta-analysis of randomized placebo-controlled trials. *CMAJ : Canadian Medical Association journal = journal de l'Association medicale canadienne.* 2006;175:377-383.

**34.** D'Souza AL, Rajkumar C, Cooke J, Bulpitt CJ. Probiotics in prevention of antibiotic associated diarrhoea: meta-analysis. *BMJ (Clinical research ed.).* 2002;324:1361.

**35.** Huang JS, Bousvaros A, Lee JW, Diaz A, Davidson EJ. Efficacy of probiotic use in acute diarrhea in children: a meta-analysis. *Digestive diseases and sciences.* 2002;47:2625-2634.

**Table S2**. Pooled estimates of probiotic’s effects on diarrhea within different subgroups.

| **Group** | **No. of comparisons** | **Pooled effect size (95% CI)** | **P-value** | **I^2^ (%)** | **P-heterogeneity** |
| --- | --- | --- | --- | --- | --- |
| **Diarrhea duration** | | | | | |
| **Type of effect size** | | | | | |
| WMD | 5 | -1.85 (-2.83, -0.86) | <0.001 | 94.7 | <0.001 |
| SMD | 14 | -0.94 (-1.32, -0.56) | <0.001 | 88.4 | <0.001 |
| **Type of probiotics** | | | | | |
| Single-strain | 12 | -1.26 (-1.76, -0.75) | <0.001 | 93.6 | <0.001 |
| Multi-strain | 7 | -0.97 (-1.52, -0.42) | <0.001 | 87.1 | <0.001 |
| **Health conditions** | | | | | |
| Acute gastroenteritis | 7 | -1.15 (-1.74, -0.55) | <0.001 | 92.1 | <0.001 |
| Acute Diarrhea | 7 | -1.69 (-2.68, -0.70) | <0.001 | 92.0 |  |
| Diarrhoeal diseases | 1 | -24.82 (-38.83, -10.81) | <0.001 | - | - |
| Acute rotavirus diarrhea | 1 | -0.41 (-0.56, -0.25) | <0.001 | - | - |
| Acute infectious diarrhea | 3 | -1.12 (-1.35, -0.89) |  | 0.0 | 0.420 |
| **Diarrhea incidence (OR)** | | | | | |
| **Type of probiotics** | | | | | |
| Single-strain | 3 | 0.46 (0.31, 0.67) | <0.001 | 96.4 | <0.001 |
| Multi-strain | 3 | 0.53 (0.12, 2.22) | 0.383 | 72.9 | <0.001 |
| **Diarrhea incidence (RR)** | | | | | |
| **Type of probiotics** | | | | | |
| Single-strain | 3 | 0.42 (0.31, 0.55) | <0.001 | 90.4 | <0.001 |
| Multi-strain | 6 | 0.61 (0.44, 0.82) | <0.001 | 0.0 | 0.611 |
